# The Use of Computed Tomography in the Study of Microstructure of Molded Pieces Made of Poly(3-hydroxybutyric-co-3-hydroxyvaleric acid) (PHBV) Biocomposites with Natural Fiber

**DOI:** 10.3390/polym13172942

**Published:** 2021-08-31

**Authors:** Wiesław Frącz, Grzegorz Janowski, Maciej Pruchniak, Łukasz Wałek

**Affiliations:** 1 Department of Material Forming and Processing, Faculty of Mechanical Engineering and Aeronautics, Rzeszow University of Technology, 35-959 Rzeszów, Poland; gjan@prz.edu.pl; 2 Department of Avionics and Control System, Faculty of Mechanical Engineering and Aeronautics, Rzeszow University of Technology, 35-959 Rzeszów, Poland; m.pruchniak@prz.edu.pl (M.P.); l_walek@prz.edu.pl (Ł.W.)

**Keywords:** CT scan, hemp fibers, biocomposites, PHBV, injection molding

## Abstract

In order to determine the structure homogeneity of biocomposites filled with fibers, as well as the evaluation of fibers’ arrangement and their orientation on the sample cross-section at varied injection rates, a study was conducted using computed tomography (CT). The main advantage of this test is the fact that in order to assess the microstructure on cross-sections, the samples do not have to be processed mechanically, which allows for presenting the actual image of the microstructure. The paper presents the issues of such tests for the biocomposite of poly (3-hydroxybutyric-co-3-hydroxyvaleric acid) (PHBV)-hemp fibers. It should be emphasized that CT scanning of PHBV-hemp fiber biocomposites is quite difficult to perform due to the similar density of the fibers and the polymer matrix. Due to the high difficulty of distinguishing fibers against the background of the polymer matrix during CT examination, a biocomposite containing 15% hemp fibers was analyzed. The samples for testing were manufactured using the injection molding process at variable injection rates, i.e., 10, 35 and 70 cm^3^/s. The images obtained by computed tomography show the distribution of hemp fibers and their clusters in the PHBV matrix and the degree of porosity on the sample cross-section. There were significant microstructural differences for the samples injected at the highest injection rates, including, among others, the occurrence of a smaller number of fibers and pores on the surface layer of the molded piece. The phenomenon observed was verified by testing chosen mechanical properties, shrinkage and water absorption of the samples. Some properties improved with an increasing injection rate, while others deteriorated and vice versa. An analysis of biocomposites’ microstructures using computed tomography provides a wide range of possibilities for future research, including an assessment of the structure of the molded parts. These tests may allow one, for example, to detect the cause of molded piece properties decreasing in a specific area as a result of a high degree of fiber disorientation, as well as the defects resulting from high porosity of the material. Such analyses can be particularly useful for producers that deal with the injection molding of pieces molded with specific properties.

## 1. Introduction

Computed tomography (CT scan) is a type of X-ray tomography that allows for obtaining spatial images by X-raying the objects examined in different directions. The advantages of this type of test are the possibilities of non-destructive testing of the internal structure of heterogeneous materials of complex shapes and thicknesses [1]. The first tomograph was built by EMI Ltd. in Great Britain in 1967, while the mathematical basis was developed by Johann Radon in 1917 [2,3]. The principle of the device’s operation is to focus the radiation beam on the object and to register its intensity. The loss of X-ray power passing through the object tested is a function of the type and thickness of the material being tested and the energy of the radiation adsorbed. The material tested is divided during the study into areas called voxels. The image obtained after digital reconstruction depends on the X-ray absorption coefficient per voxel belonging to the tested material layer [4]. For the testing of polymer composites, high accuracy of mapping the internal structure of the analyzed specimen is necessary in order, among other thing, to study the structure of the material and perform measurements of inclusion geometry, defects and microstructural defects [5].

The CT scan, as was mentioned previously, is a non-destructive test, as the test sample is not destroyed in the process and its inner structure is not deformed in the element’s machining process during preparation for the test. The primary advantage of this examination method is the ability to evaluate both the number and size of pores occurring in the structure. It is also possible to examine the distribution of pores based on their size. This method is characterized by high precision, and its described capabilities could be applied not only in porosity examination, but also in determining the distribution and orientation of fibers used as the admixture in the composite material. Despite its numerous advantages, the CT scan, as any other method, has its disadvantages. One of the main disadvantages is undoubtedly the high price of the devices necessary to perform the tests and, as it is a specialized piece of equipment, the necessity of conducting tests in the presence of specialized personnel. Experienced staff are also invaluable at the stage of analyzing the collected data and their appropriate reduction and processing in order to create the final image to visualize the internal structures of the material, both in 2D form for a specific cross-section and in 3D form for the area under consideration. The stage of data processing and generating visualizations requires high processing power in the computers used [5].

Moreover, the porosity measured with the CT scanner underestimates the composite porosity because it is not able to take into account voids smaller than the resolution in diameter. Many voids at the fiber-matrix interfaces may also not be detected using this method [6].

Computed tomography studies are widely used in the analysis of porous polymer material structures [7], as well as with fillers in the form of mats, fabrics [8] and short fibers [9,10]. In the case of testing the composites of the thermoplastic matrix filled with synthetic fibers, the receiving image will provide satisfactory results because of the relatively high test accuracy resulting from large differences between the fibers and matrix density. In the case of composites filled with plant-derived fibers, computed tomography tests are much more difficult to perform, which results from the lower density of these fibers compared to synthetic fibers (Table 1), and a similar density to the polymer matrix [11,12,13]. It should also be noted that the plant-origin fibers are heterogeneous, built of components such as cellulose, hemicellulose, and lignin with different content. (Table 2) [11,12,13]. The hydroxyl (OH) groups in cellulose, hemicellulose and lignin build up a large number of hydrogen bonds inside the macromolecule and between macromolecules in the cell wall of plant fibers, which affects the hydrophilic properties of the fibers and, as a consequence, the presence of water in the porous structures of natural fibers [14,15,16,17]. Due to these factors, testing the microstructure of thermoplastic polymer composites filled with fiber fillers is difficult to perform. In turn, the analysis of the microstructure of injection molded pieces from this type of composite is very desirable due to the great interest in this type of filler as an interesting substitute for synthetic fillers.

One of the interesting biocomposites, the study of the microstructure of which is very desirable but problematic, is biocomposites based on polyhydroxybutyrate-co-valerate (PHBV) filled with plant origin fibers [18,19,20].

In recent years, there has been a large increase in the use of natural fibers to produce new types of environmentally friendly composites. In the case of plant fibers, due to their wide availability in the natural environment, their cost as a raw material is relatively low, and so they can compete with synthetic fibers, such as fiberglass. In addition to the fact that these materials are of natural origin and are fully biodegradable, they are characterized by a low density while maintaining high strength and rigidity [21,22].

Pure PHBV, which is a biopolymer belonging to the group of polyhydroxyalkanoates, has similar properties to polypropylene, is of natural origin and is biodegradable. Unfortunately, due to high manufacturing costs, there is a problem with its commercialization as a material for the production of, among other things, injection-molded pieces [18,19,23]. In order to reduce production costs and improve mechanical properties, a new biocomposite consisting of PHBV as a matrix and a filler in the form of short hemp fibers was prepared. The biocomposite produced has relatively better properties compared to pure PHBV, and its production costs are lower [18].

An analysis of the microstructure of PHBV matrix biocomposites filled with hemp fibers using computed tomography can provide broad possibilities for future research, including an assessment of the structure of the areas of molded pieces obtained in cross-sections. These tests may allow, for example, for detecting the reason for the deterioration of the properties of the molded pieces in a specific area as a result of a high degree of fiber disorientation, as well as defects resulting from the high porosity of the material. Such analyses can be particularly useful for manufacturing companies specializing in the injection molding process of products with specific properties. Hence, the purpose of this work is to analyze the microstructure of molded pieces from the PHBV-hemp fiber biocomposite using computed tomography, depending on the variable injection rate using during the manufacturing of specimens.

## 2. Techniques and Procedures

### 2.1. Materials

As the polymer matrix, PHBV with the Enmat Y1000 trade name of Helian Polymers (Belfeld, The Netherlands) in powder form was used. The density of the biopolymer was 1250 kg/m^3^, and the melting temperature ranged from 165 to 175 °C [24,25].

As the filler in the polymer matrix, hemp fibers supplied by EKOTEX company (Kowalowice, Poland) were applied. The average ratio of length (L) to diameter (d) of the fibers was about 10 (for L = 1 mm). The biocomposite was prepared where the mass content of fibers in the polymer matrix was 15%.

### 2.2. Composite Preparation

The PHBV-hemp fiber biocomposite was produced by means of an extrusion process using a ZAMAK EHP-25E single screw extruder (produced by ZAMAK Mercator company, Skawina, Poland), at a screw speed of 100 rpm. The machine settings of the plasticization unit in which the biocomposite was produced are presented in Table 3. It should be noted that both the polymer matrix and hemp fibers were dried before the extrusion process for 3 h at 90 °C.

To produce the samples, the DrBoy 55E injection molding machine produced by BOY Maschines Inc. (Exton, PA, USA) equipped with a Priamus data acquisition and processing system (by Priamus System Technology, Rheinweg, Switzerland) to monitor and control the injection molding process was used. The laboratory injection mold with two cavities and changed inserts was used for the manufacturing of the uniaxial tensile test samples in accordance with PN-EN ISO 527-1 [26]. The processing parameters of the injection molding process are shown in Table 4. Three groups of injected samples were obtained for a variable injection rate of 10, 35, and 70 cm^3^/s.

### 2.3. The Composite Structure Examination Using CT

**Figure 1:**
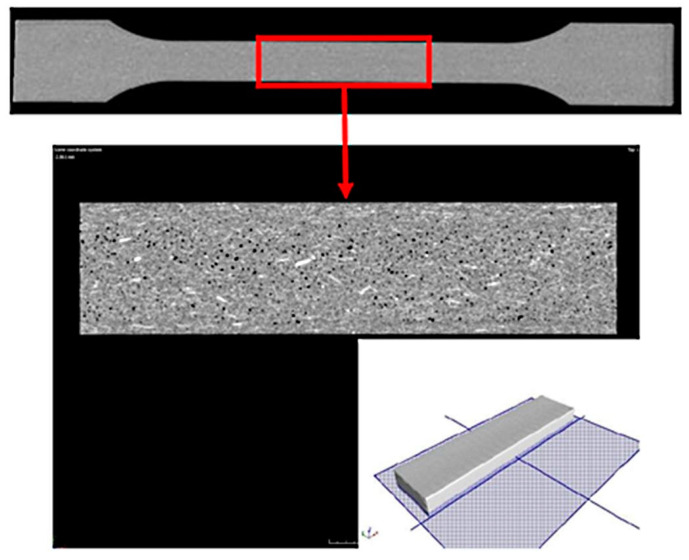
Sample test result using computed tomography with a resolution of 170 µm and a chosen area of the test sample made with a resolution of 50 µm with imaging of the test plane on the cross-section.

Computed tomography tests were performed for the samples prepared from the biocomposite tested. For this purpose, the tests were carried out using the CT METROTOM 1500 system. The tests were commissioned to be performed by Laboratory of Reverse Engineering at the Wroclaw University of Science and Technology Wroclaw, Poland). The measurement parameters of the samples are shown in Table 5. The microstructure of the manufactured specimens at a variable injection rate of 10, 35 and 70 cm^3^/s was examined. Two research tests were made to conduct the analysis. The first test was made with a resolution of 170 µm, which allowed for the entire sample to be analyzed. The second test was made with a resolution of 50 µm, which allowed for testing a smaller area of the samples. The chosen area of the specimen for the uniaxial tensile test on its cross-section (in the middle of specimen thickness) was analyzed (Figure 1). The dimensions of the area tested at a resolution of 50 µm were 40 mm × 10 mm × 4 mm. Only the test with a 50 µm voxel size allowed for determining the diameter and volume of fibers and pores registered for samples tested.

## 3. Results and Discussion

### 3.1. The Biocomposite Microstructure Assessment

**Figure 2:**
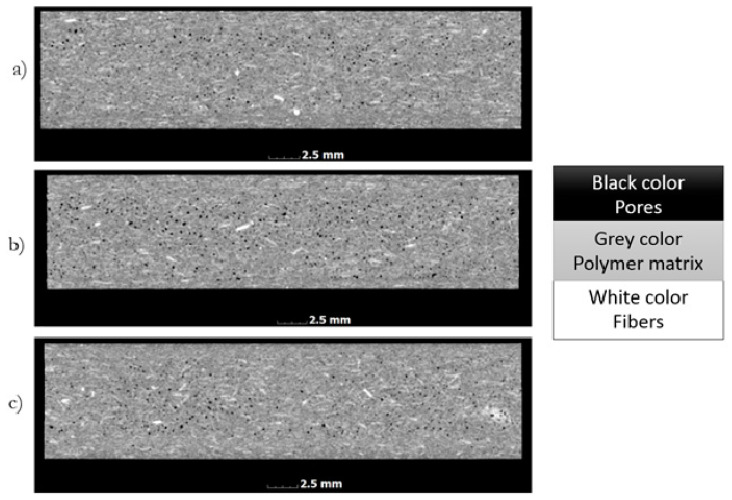
Images of samples based on their cross-sections, formed at a variable injection rate: (**a**) 10, (**b**) 35, and (**c**) 70 cm^3^/s.

**Figure 3:**
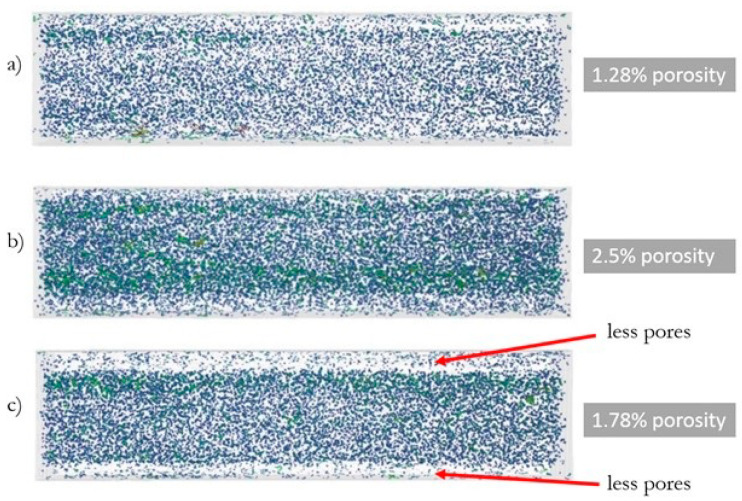
The results of the porosity analysis for injected samples at a variable injection rate: (**a**) 10, (**b**) 35, and (**c**) 70 cm^3^/s—digital reconstruction.

**Figure 4:**
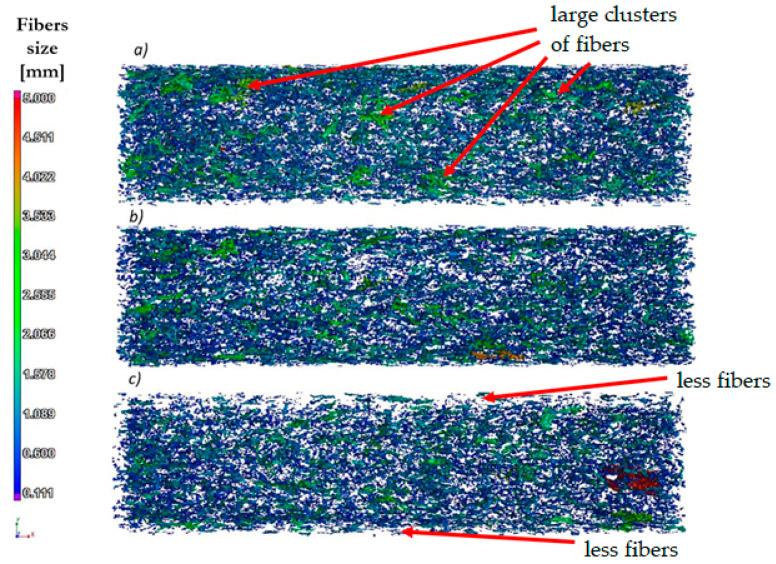
The results of the fibers and their clusters orientation analysis for injected samples at a variable injection rate: (**a**) 10, (**b**) 35, (**c**) and 70 cm^3^/s—digital reconstruction.

Computed tomography studies allow for spatial geometry to analyze physical objects, where the gray scale recorded for the object is proportional to its density (Figure 2). The polymer matrix of samples has a slightly different density than fibers. The difference recorded in the shades of gray allows for the threshold value to be set and, on this basis, to separate the gray scale for fibers and matrix. As a result of such thresholding, fiber geometry is obtained, which can then be analyzed, e.g., by determining the sphere describing the geometry obtained. Figure 2 also shows actual sample images (for a 50 µm voxel size) after computed tomography. Black objects indicate pores, white objects indicate fibers, and gray objects indicate the polymer matrix. Due to the small differences in the matrix and fiber density, a small number of fibers embedded in the polymer matrix are noticeable. In order to obtain more accurate results, the images were digitally reconstructed, where the results are shown in Figure 3 and Figure 4.

The following porosities were obtained (Figure 3) for samples injected with variable rates: 10 cm^3^/s—1.28% porosity, 35 cm^3^/s—2.5% porosity, and 70 cm^3^/s—1.78% porosity. In the case of the specimen injected at a rate of 70 cm^3^/s, an irregular distribution of pores in the matrix is noticeable—an increased number of pores are in the core of the sample, and significantly fewer are observed at the top layer.

A partial analysis of fibers’ orientation on the sample sections was made (Figure 4). For samples injected at the lowest speed (10 cm^3^/s), there is a tendency for the fibers to clump together into larger clusters. For the sample injected at the highest speed (70 cm^3^/s), the fibers have the lowest tendency to form larger clusters; in addition, the phenomenon of very small amounts of fibers was observed at the surface layer of the sample.

**Figure 5:**
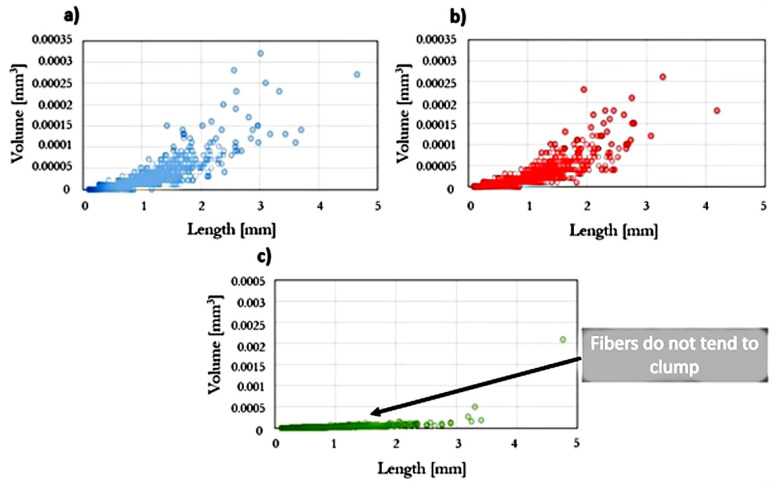
The volume of fibers and their clusters as a function of their length for injected samples at variable injection rate: (**a**) 10, (**b**) 35, and (**c**) 70 cm^3^/s.

Statistical analysis was performed on the relationship between the volume of fiber aggregates and their length (Figure 5). It was noted that for the composite injected at the highest rate, the fiber clusters have the smallest dimensions. In addition, using the software, the mass fraction of fibers was determined to be 12%, where the real mass fraction of fibers in the prepared biocomposite was 15%. It is believed that this is due to the structure of natural fibers, including the different density of their individual components.

### 3.2. Veryfication Tests

**Figure 6:**
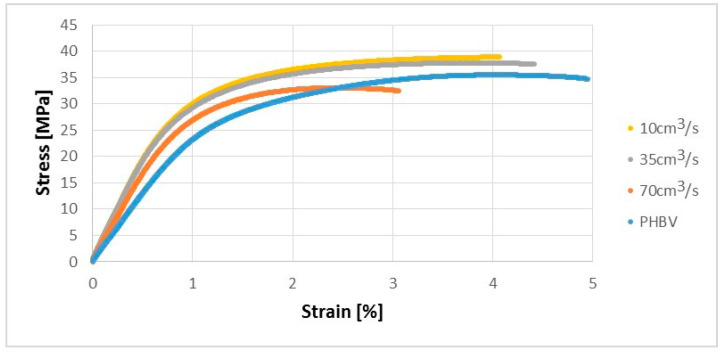
Stress-strain characteristics for tested samples produced at a variable injection rate compared to a pure PHBV sample produced at 35 cm^3^/s.

Due to the interesting results regarding the microstructural analysis of samples manufactured at a variable injection rate, the verification tests were carried out to confirm the phenomena observed. The Zwick Z030 testing machine produced by the ZWICK ROELL Group (Ulm, Germany) was used to determine the strength properties of the biocomposites obtained. The uniaxial tensile test was carried out in accordance with PN-EN ISO 527-1 for the injected samples. On the basis of the uniaxial tensile test, one can say that the mechanical properties of the injected samples were improved at 10 and 35 cm^3^/s in terms of tensile strength (by approx. 9% and 6%) compared to pure PHBV. In turn, for the biocomposite injected at the highest speed (70 cm^3^/s), a decrease in the tensile strength value by about 7% compared to pure PHBV was received (Figure 6).

Undoubtedly, the noticed differences in the structure of biocomposites will also affect their other mechanical properties and performances. A similar trend was noted to test hardness and the Charpy impact strength. The tests were carried out in accordance with PN-EN ISO 2039-1 [27] using a Zwick 3106 hardness tester. The Charpy impact strength tests were carried out in accordance with PN-EN ISO 8256 [28]. The CAEST 9050 impact pendulum was used for this purpose.

**Figure 7:**
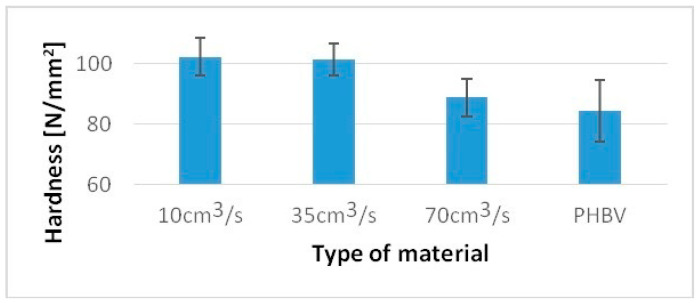
Hardness of tested samples produced at a variable injection rate compared to a pure PHBV sample produced at 35 cm^3^/s.

**Figure 8:**
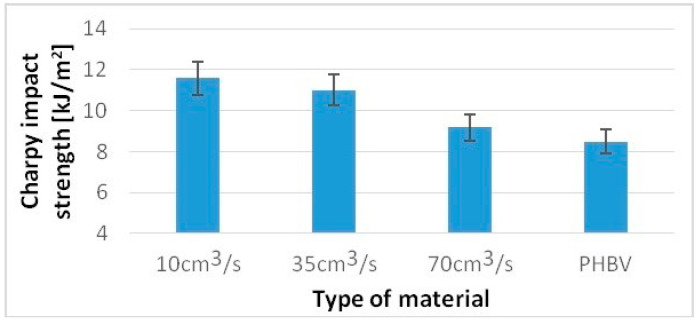
The Charpy impact strength of tested samples produced at a variable injection rate compared to a pure PHBV sample produced at 35 cm^3^/s.

For a sample injected at a rate of 10 cm^3^/s, a 36% higher value of the Charpy impact strength was obtained, while for samples injected at a speed of 70 cm^3^/s, only an 8% increase compared to pure PHBV was obtained (Figure 7). For samples injected at a speed of 10 cm^3^/s, 20% higher hardness was obtained, and for samples injected at a speed of 70 cm^3^/s, only a 5% increase in hardness was obtained compared to pure PHBV (Figure 8).

**Figure 9:**
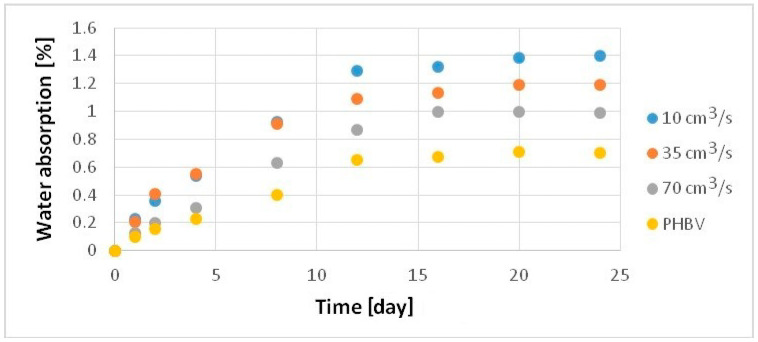
Water absorption of samples produced at a variable injection rate compared to a pure PHBV sample produced at 35 cm^3^/s.

The water absorption of the samples produced was studied following the standard PN-EN ISO 62 [29]. Analyzing the results (Figure 9), a significant increase in water absorption was observed for samples of the composites filled with hemp fibers. From the first days of the measurement, a moderate increase in water absorption is noticeable (relative to the sample from pure PHBV) for the sample made with an injection rate of 70 cm^3^/s and a significant increase for the samples manufactured with injection speeds of 30 and 10 cm^3^/s. After 24 days of testing, water absorption of the biocomposite sample manufactured at injection rate of 70 cm^3^/s rose by 41% relative to the pure PHBV sample, 69% for the sample manufactured at a speed of 35 cm^3^/s, and over 100% for the sample injected at a speed of 10 cm^3^/s.

**Figure 10:**
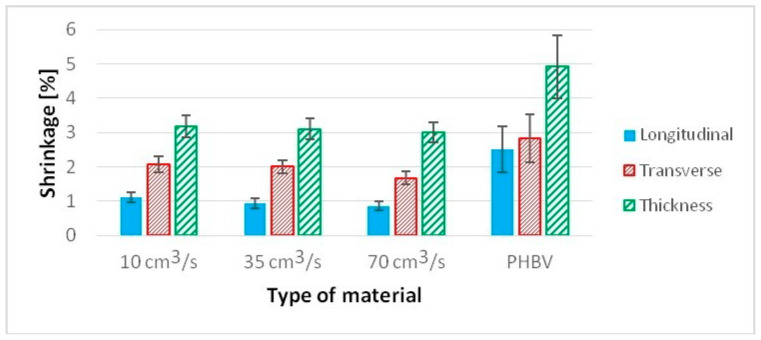
Linear shrinkage of samples produced at a variable injection rate compared to a pure PHBV sample produced at 35 cm^3^/s: longitudinal, transverse, in thickness.

The shrinkage of specimens with “dogbone” geometry was tested, taking into account the standard PN-EN ISO 294-4 [30]. The presence of hemp fibers (15%) in the composite significantly reduces shrinkage in comparison to pure PHBV (Figure 10). This is related to the reduction in shrinkage in the longitudinal direction in particular, where, depending on the injection rate, a decrease in this property ranges from 56.2% to 65.8% in comparison to pure PHBV, i.e., with an increasing injection rate, the shrinkage of molded pieces in the longitudinal direction decreases. Smaller shrinkage reduction can be observed in other measurement directions. In the case of transverse shrinkage, the reduction in this parameter ranges from 26.4% to 40.9% in comparison to pure PHBV. In this case, property improvement is also tied with an increase in the injection rate, while changing the injection rate from 35 to 70 cm^3^/s results in the reduction in transverse shrinkage by 11.7%. In the molded piece, thickness shrinkage reduction ranges from 35.2% to 38.7%. In this case, the decreasing value of this type of primary shrinkage is also observed with the increase in injection rate.

## 4. Discussion

The CT scan is undoubtedly a good modern technique for assessing the microstructure of polymers undertaken by researchers primarily with the aim of assessing the presence of porous structures.

In one previous study [8], the authors paid attention to the possibility of the coexistence of two or more 3D structures inside the test sample. The authors also, as in the case of the presented work, dealt with a material with a heterogeneous internal structure—as was the case for the sample injected at a speed of 70 cm^3^/s. These types of anomalies are usually the source of the heterogeneity of materials. Additionally, the article proposed a method of mathematical description of the presence of pores depending on their number and size. The presented double Gauss model could also be used in the description of the pore distribution in the biocomposite discussed in this paper. For a better examination of the internal structures of the tested samples, as in the case of polyurethane foams, it may be recommended to perform more advanced tests, such as SEM, gas pycnometry and SAXS.

In another article [31], the authors discussed, inter alia, the issue of the functionality of pores present in the structures of manufactured products. They found that high porosity may be a desirable characteristic in certain applications. The authors used the example of medicine and bone loss supplementation, where the ability of the composite used to pass nutrients through its interior is indicated. Similarly, in the case of the biocomposite with hemp fibers considered in this paper, it is possible to find applications that will also favor products with high permeability, for example the ability to drain water from its surface. It should be noted that a higher porosity results in less material consumption for products of the same volume. This is an advantage related to the reduction in the financial costs of production.

Computed microtomography can also be used to assess fiber orientation. The authors another previous article [32] presented, among others, the results of their research and analysis of the influence of glass fiber admixtures on the properties of the tested composite with the use of PLA and PMMA. During tests of mechanical properties, a significant increase in the strength of the material with the addition of glass fibers was observed. The increase in Young’s modulus by approx. 210% in the longitudinal direction of the fiber orientation in the composite structure. A similar tendency to improve mechanical properties can be noticed in the tested biocomposites with hemp fiber filler.

In turn, in another article [33], the authors discussed the issues of controlling the orientation of natural fibers, i.e., cellulose ones, in the PLA matrix by controlling the processing parameters of product manufacturing. The key factors responsible for the control of the fiber orientation include such factors as the rheological properties of the material, thermal expansion of fibers and polymers, temperature distribution in the plasticizing system and nozzle geometry. The research carried out on a biocomposite with hemp fibers clearly shows that the flow rate of the polymer into the mold is an equally important factor. It can significantly change the properties of the products, as well as causing changes in the spatial structures of its interior microstructure.

The obtained test results in this presented work can confirm the distribution of the fibers and pores in the sample for injected samples at speeds of 10 and 35 cm^3^/s—the samples made from biocomposites have similar mechanical properties, significantly better than pure PHBV. In turn, for the samples injected at a speed of 70 cm^3^/s, mechanical properties similar to pure PHBV were noted, and significantly worse than the samples of biocomposites injected at lower injection rates. This decrease in the mechanical properties may be due to the noticeably uneven distribution of fibers and pores on the cross-sections of the sample, as observed by using computed tomography. Pores and fibers accumulate in the core, while only the presence of pure PHBV is visible at the top layer of the molded pieces. The obtained phenomenon may result from high shear stress near the walls when filling the forming cavity in the injection process, which may reduce the viscosity of the polymers moving at the walls of the forming cavity. Then, as a result of the temperature gradient between the surface of the injection mold and the polymer, the material solidifies at the walls of the cavity, while the core of the material stream with a large amount of fibers is still in a plasticized state. The phenomenon observed is an undesirable effect because the material is heterogeneous in the entire volume of the molded pieces. In the case of the injection molded pieces, there is a tendency towards an uniform distribution of fibers in a polymer matrix in order to reduce the areas of molded parts with significantly worse mechanical properties and visual defects. On the other hand, the presence of the PHBV matrix at the top layer in relation to the small number of fibers and pores (Figure 3c and Figure 4c) for the composite injected at a speed of 70 cm^3^/s results in the lowest water absorption and processing shrinkage compared to other composites injected at a lower speed.

Therefore, the above results indicate that in order to properly produce specimens with a dog-bone geometry from a PHBV-hemp fiber biocomposite, an injection speed of 10 cm^3^/s should be used to maximize the mechanical properties. On the other hand, to reduce the water absorption rate and shrinkage, an injection speed of 70 cm^3^/s should be used.

## 5. Conclusions

With the use of a CT scan, the results obtained show the location of hemp fibers, their aggregates in the PHBV matrix and the degree of porosity in the cross-sections of the part of molded pieces. The main advantage of this test is the fact that in order to assess the microstructure on cross-sections, the samples do not have to be destructively machined, which allows for presenting a real image of the microstructure.

It was noted that the examined mass content of fibers in the polymer matrix by computed tomography was about 12% (the real value was about 15%). The porosity of the composites obtained was also assessed. It was noted that the porosity was in the range of 1.28% to 2.5%.

It should be emphasized that computed tomography of the PHBV-hemp fiber biocomposite is quite a difficult test to perform due to similar fiber and polymer matrix densities. Therefore, in order to obtain the most accurate image, a sampling resolution of 50 μm was selected.

It is difficult to determine the orientation of individual fibers using computed tomography. This is due to the fact that the fiber and matrix densities are similar, as well as the tendency of fibers to aggregate.

In order to verify the results obtained from computed tomography, the samples were tested to determine the mechanical properties and performance properties (water absorption and shrinkage of the samples). In the case of biocomposites injected at speeds of 10 and 35 cm^3^/s, better mechanical properties were obtained compared to pure PHBV. In the case of the composite injected at a speed of 70 cm^3^/s, significantly worse mechanical properties, but lower water absorption and shrinkage of samples were obtained compared to biocomposites injected at lower speeds.

Significant microstructural changes were noted for the samples injected at the highest injection rate (70 cm^3^/s). The phenomenon was the presence of a smaller number of fibers and pores near to the surface layer of the molded piece. This phenomenon may justify the obtaining of worse mechanical properties and better performance properties of the injected samples at the highest rate. It was also noted that the samples injected at a rate of 70 cm^3^/s have a microstructure where the fibers do not tend to clump into larger aggregates.

## Figures and Tables

**Table 1:** Physico-chemical properties of some fibers used as fillers in polymer matrix [11,13].

Fiber Source	Density (kg/m^3^)	Elongation (%)	Tensile Strength (MPa)	Young’s Modulus (GPa)
Flax	1500	2.7–3.2	345–1035	27.6
Hemp	1500	1.6	690	70
Kenaf	1500	1.3–5.5	195–666	60–66
Coniferous tree	1500	–	1000	40
Bamboo	1400–1450	2.5–3.7	140–800	11–32
Glass fiber type E	2500	2.5	2000–3500	70
Glass fiber type S	2500	2.8	4570	86

**Table 2:** Mass content of individual components for plant fibers [11,14].

Fiber Type	Cellulose (wt.%)	Hemicellulose (wt.%)	Lignin (wt.%)	Others (wt.%)
Flax	71	18.6–20.6	2.2	1.5
Kenaf	72	20.3	9	–
Bamboo	26–43	30	21–31	–
Jute	61–71	14–20	12–13	0.5
Hemp	68	15	10	0.8
Deciduous trees	44 ± 3	32 ± 5	18 ± 4	0.2–0.8
Coniferous tree	42 ± 2	26 ± 3	29 ± 4	0.2–0.8

**Table 3:** Temperature set of a single-screw extruder for the PHBV-hemp fiber biocomposite.

Temperature [°C]				
Head	Zone 3	Zone 2	Zone 1	Feed Hopper
170	165	155	145	35

**Table 4:** Processing parameters of injection molding for molded pieces.

Parameter	Value
Mold temperature [°C]	60
Melt temperature [°C]	167
Cooling time [s]	25
Packing time [s]	25
Packing pressure [MPa]	30
Injection speed [cm^3^/s]	10, 35, 70

**Table 5:** Projection and reconstruction parameters of the biocomposite microstructure image using computed tomography.

Projection Parameters		Reconstruction Parameters
Measurement 1	Measurement 2	Measurement for Voxel Size: 50 µm
Voltage: 170 kV	Voltage: 100 kV	Number of projections: 1050
Current: 300 µA	Current: 200 µA	Reconstruction algorithm: Feldkamp
Voxel size: 170 µm	Voxel size: 50 µm	Correction of circular artifacts every 10°
Pre-filtration: 0.25 mm	Pre-filtration: none	Noise filtration: Sheep_Logan
Detector: - enhancement: 8× - integration time: 1 s	Detector: - enhancement: 8× - integration time: 1 s	Offset correction: - number of averaged images: 10
Temperature: 20.8 °C	Temperature: 20.8 °C	Detector gain correction: - homogenization steps: 7 - number of averaged images: 10

## Data Availability

Not applicable.
